# Acute Impact of Pacing at Different Cardiac Sites on Left Ventricular Rotation and Twist in Dogs

**DOI:** 10.1371/journal.pone.0111231

**Published:** 2014-10-23

**Authors:** Zhi-Wen Zhou, Bu-Chun Zhang, Yi Yu, Kai Guo, Wei Li, Rui Zhang, Peng-Pai Zhang, Yi-Gang Li

**Affiliations:** 1 Department of Cardiology, Xin Hua Hospital Affiliated to Shanghai Jiao Tong University School of Medicine, Shanghai, People's Republic of China; 2 Department of Cardiology, Shanghai Xuhui Central Hospital, Shanghai, People's Republic of China; 3 Department of Cardiology, The Affiliated Hospital of Xuzhou Medical College, Jiangsu province, People's Republic of China; Scuola Superiore Sant'Anna, Italy

## Abstract

**Objectives:**

We evaluated the acute impact of different cardiac pacing sites on two-dimensional speckle-tracking echocardiography (STE) derived left ventricular (LV) rotation and twist in healthy dogs.

**Methods:**

Twelve dogs were used in this study. The steerable pacing electrodes were positioned into right heart through the superior or inferior vena cava, into LV through aorta across the aortic valve. The steerable pacing electrodes were positioned individually in the right atrium (RA), right ventricular apex (RVA), RV outflow tract (RVOT), His bundle (HB), LV apex (LVA) and LV high septum (LVS), individual pacing mode was applied at 10 minutes interval for at least 5 minutes from each position under fluoroscopy and ultrasound guidance and at stabilized hemodynamic conditions. LV short-axis images at the apical and basal levels were obtained during sinus rhythm and pacing. Offline STE analysis was performed. Rotation, twist, time to peak rotation (TPR), time to peak twist (TPT), and apical-basal rotation delay (rotational synchronization index, RSI) values were compared at various conditions. LV pressure was monitored simultaneously.

**Results:**

Anesthetic death occurred in 1 dog, and another dog was excluded because of bad imaging quality. Data from 10 dogs were analyzed. RVA, RVOT, HB, LVA, LVS, RARV (RA+RVA) pacing resulted in significantly reduced apical and basal rotation and twist, significantly prolonged apical TPR, TPT and RSI compared to pre-pacing and RA pacing (all *P*<0.05). The apical and basal rotation and twist values were significantly higher during HB pacing than during pacing at ventricular sites (all *P*<0.05, except basal rotation at RVA pacing). The apical TPR during HB pacing was significantly shorter than during RVOT and RVA pacing (both *P*<0.05). The LV end systolic pressure (LVESP) was significantly lower during ventricular pacing than during pre-pacing and RA pacing.

**Conclusions:**

Our results show that RA and HB pacing results in less acute reduction on LV twist, rotation and LVESP compared to ventricular pacing.

## Introduction

Ventricular pacing could induce abnormal, asynchronous electrical activation and mechanical motion of the left ventricle (LV) [1], [2]. Previous studies suggested that permanent ventricular pacing might induce LV desynchronization and negatively affect LV function in patients with sick sinus syndrome and in patients with normal baseline QRS duration [2], [3].

Cardiac pacing site might be an important determinant for pacing-induced changes on LV mechanical motion, and pacing at some specific cardiac sites might minimize cardiac pacing-induced unfavorable effects [4], [5]. Because of the 3-dimensional nature of cardiac structure and motion, the cardiac pacing-induced LV mechanical change is complex, which involves not only the myocardium at systolic and diastolic phases, but also affect other myocardial moving, such as twist and rotation [6], [7].

LV twist and rotation are important elements of cardiac mechanics [7]. It has been proposed that LV twist and rotation are sensitive markers reflecting LV regional and global function changes [7], [8]. The purpose of this study was to evaluate the acute impact of pacing at different cardiac sites on LV twist and rotation derived from speckle-tracking echocardiography in healthy dogs.

## Methods

### Ethics Statement and Animal Model Preparation

All animal protocols in this study were approved by the Animal Care and Use Committee, Research Institute of Medicine, Shanghai Jiao Tong University, in accordance with National Institutes of Health guidelines and public law. All surgery was performed under sodium pentobarbital anesthesia, and all efforts were made to minimize suffering.

Animals (8 male and 4 female dogs, weight: 20.2±1.5 Kg) were purchased from Shanghai Jiagan biological technology CO., LTD (168 Xiannong Road, Pujiang Town, Shanghai, People's Republic of China). The study was performed in a cardiac catheterization laboratory equipped with fluoroscopy (Innova 3100, GE Medical Systems) and electrophysiology catheters (Bard EP Recording System, Glens Falls Operation division of C.R. Bard, Inc., USA). After 12 hours fasting, dogs were anesthetized with sodium pentobarbital (25–25 mg/kg induction, followed by 1.0 mg/kg/h intermittent bolus injectionas needed). The 6F sheaths were inserted percutaneously into the femoral vein and artery for venous and arterial access. The external jugular vein and/or artery were prepared if more venous and/or arterial accesses are needed.

With the help of fluoroscopy and an ultrasound system (Vivid 7, GE Medical Systems), the fluid filled 5F pigtail catheter (Cardis, Johnson & Johnson Medical Shanghai, LTD) was placed in the LV across the aortic valve to monitor LV pressure during the whole experimental procedure. The steerable pacing electrodes were positioned into right heart through the superior or inferior vena cava, into LV through aorta across the aortic valve via femoral or carotid artery. The steerable pacing electrodes were positioned individually in the right atrium (RA), right ventricular apex (RVA), RV outflow tract (RVOT), His bundle (HB), LV apex (LVA) and LV high septum (LVS) for pacing (≥5 min) under fluoroscopy and ultrasound guidance, as previously described [5], [9], [10]. RARV pacing was performed by pacing at RA and RVA with atrioventricular synchrony (70–120 ms A–V interval, as indicated) using dual chamber temporary pacemaker (Medtronic 5388, Medtronic Inc., USA). For HB pacing, the pacing electrode was positioned at the tricuspid annulus, close to atrial septum, where the HB electrogram was recorded.

### Echocardiography Acquisition and Pressure Monitoring

Individual pacing mode was applied at 10 minutes interval. After at least 5 minutes pacing under stabilized hemodynamic conditions, frame rate, sector width and depth were manually adjusted to achieve a quality image with frame rates from 60 to 80 frames per second using the Vivid 7 ultrasound machine equipped with 3.5-MHz transducer (GE Medical Systems). Parasternal LV basal (mitral valve level) and apical short-axis images were acquired from three consecutive cardiac cycles. Apical 4 chamber views were also obtained. LV end diastolic volume (LVEDV), LV end systolic volume (LVESV) and LVEF was measured using Simpson’s biplane method. The Maximal annular plane systolic excursion of both mitral (MAPSE) and tricuspid (TAPSE) were also measured by placing the M-mode cursor oriented to the junction of the mitral annular plane with the LV lateral wall as well as tricuspid annular plane with the RV free wall using images of the apical four-chamber view [11]. The QRS duration was measured by using Bard EP Recording System. LV end-diastole was defined at the peak of the R wave on the electrocardiographic QRS complex which signals the depolarization of the ventricle and the end-systole defined as the end of T-wave from ECG [12]–[15].

### LV Rotation and Twist Assessment

**Figure 1:**
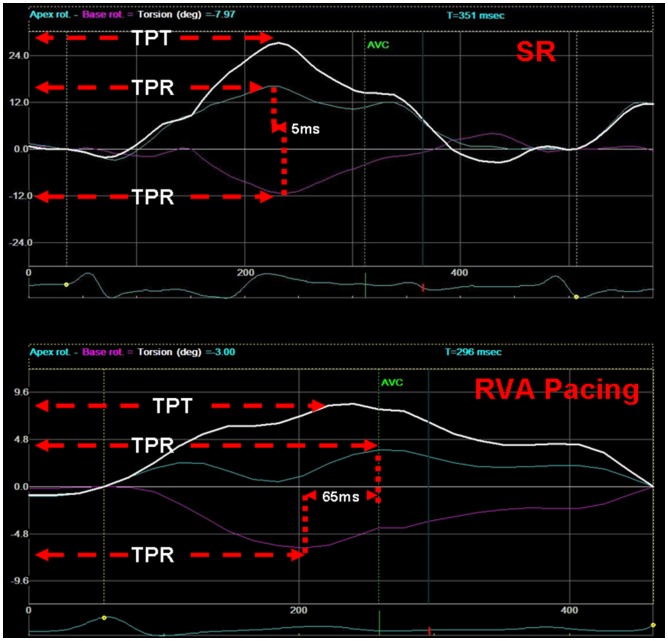
Representative image of SR and RVA pacing for LV rotation and twist in the same dog. As shown by the graph, the degree of rotation and twist was smaller at RVA pacing than SR, and the apical TPR, RSI, and TPT of RVA pacing were longer compared to SR. SR: sinus rhythm; RVA: right ventricular apex; TPR: time to peak rotation; TPT: time to peak twist; RSI: rotational synchronization index.

Analysis of echo images was performed offline on a Windows workstation using a customized software package (EchoPAC, ver. 9.0, GE Medical Systems) and observer was blinded to the pacing mode. A circular region of interest (ROI) was specified between endocardial and epicardial borders, at the reference end diastolic frame of the image loop. An automated tracking algorithm was used to track the speckles within the specified ROI throughout the cardiac cycle. This system follows acoustic markers in the myocardium in the subsequent frames of the image loop during a cardiac cycle and computes time-rotation and -twist curves automatically. The peak rotation and twist values were then acquired from the curves. The Q-wave to peak rotation interval (time to peak rotation, TPR) was defined as the interval from the beginning of the Q-wave to the peak apical counter-clockwise or peak basal clockwise rotation, and the Q-wave to peak twist interval (time to peak twist, TPT) defined as the interval from the beginning of the Q-wave to the peak twist (Figure 1). Apical-basal peak rotation delay was calculated by subtracting the basal Q to peak rotation interval from apical one as the representative rotational synchronization index [RSI = apical TPR (aTPR) – basal TPR (bTPR)] (Figure 1) [16].

To minimize the affection of heart rate, TPR, TPT, and RSI data were adjusted by R–R interval as follow: TPR/R-R interval, TPT/R-R interval, and RSI/R-R interval.

### Statistical Analysis

All data are expressed as means ± standard deviation. Data were analyzed by one-way analysis of variance (ANOVA) followed by post hoc Fisher's least significant difference test. The relation among rotation and twist with LVEDP, LEVSP, LA, LVEDV, LVESV, LVEF, MAPSE, TAPSE, QRS duration, TPT and RSI was assessed with Pearson correlation analysis. Inter-observer variability of measurements was determined by two independent blinded observers on 25 randomly selected cases for twist and TPT measurements.

Analyses were performed using the SPSS 14.0 statistical software (SPSS, Chicago, IL, USA). All statistical tests were two sided, and a *P* value <0.05 was considered to indicate statistical significance.

## Results

Twelve dogs were used in this study. Anesthetic death occurred in 1 dog and another dog was excluded because of bad imaging quality. Data from10 dogs were analyzed, and all obtained images were suitable for analyzing STE. The pre-pacing heart rate was 116±5 bpm, and increased equally post-pacing (all *P*<0.001). The pacing rates were similar among all pacing groups (Table 1).

**Table 1:** Parameters of TPT, TPR and RSI adjusted by the R–R interval, echocardiography, MAPSE, TAPSE, left ventricular pressure and heart rate at SR and pacing.

Pacing								
	SR	RA	HB	RVOT	RVA	RVRA	LVS	LVA
HR(beats/min)	116±5	125±7*	126±7*	128±7*	126±8*	129±8*	127±8*	129±9*
LVEDV(ml)	62.2±9.4	60.1±6.2	59.6±8.1	57.7±7.1	59.2±5.4	60.0±4.2	58.4±6.3	58.9±4.1
LVESV(ml)	28.6±3.9	25.6±3.4	24.9±4.2	27.1±4.1	26.3±3.5	27.8±4.2	25.3±5.3	26.7±4.0
LVEF(%)	59±3.5	55±3.9	55±5.2	54±6.2	55±4.9	56±4.0	58±4.2	57±4.6
MAPSE(mm)	6.3±1.1	6.0±1.0	6.1±1.3	6.1±1.2	6.2±1.1	6.1±1.0	5.9±1.3	6.1±1.2
TAPSE(mm)	6.5±1.5	6.8±1.7	6.6±1.2	6.5±1.7	6.6±1.6	6.4±1.4	6.4±1.7	6.8±1.6
LVESP(mmHg)	86.1±9.5	82.3±7.2	78.4±5.5*^†^	77.3±9.8*^†^	76.7±6.7*^†^	78.2±5.1*^†^	75.9±5.0*^†^	76.3±4.9*^†^
LVEDP(mmHg)	5.4±2.1	5.7±3.2	6.3±3.0	6.1±4.3	5.7±2.6	6.2±3.1	6.0±2.4	5.5±2.9
aTPR/R-R (%)	41.5±7.0	48.1±6.5	59.0±6.8*^†^	64.3±8.5*^† ‡^	64.5±8.4*^† ‡^	62.0±6.2*^†^	61.7±12.0*^†^	64.4±9.2*^†^
bTPR/R-R (%)	44.8±8.7	48.9±9.1*	48.3±5.4*	52.4±6.7*	50.8±9.2*	55.7±9.1*^‡^	54.0±7.8*	55.2±8.3*^‡^
TPT/R-R (%)	43.2±7.0	50.2±7.3	60.0±7.2*^†^	60.7±9.0*^†^	66.0±8.9*^†^	64.2±8.2*^†^	63.0±10.1*^†^	63.3±8.9*^†^
RSI/R-R (%)	−3.3±5.5	0.7±7.3	10.7±6.7*^†^	12.0±6.0*^†^	13.7±5.7*^†^	6.3±9.5*^†^	7.7±8.1*^†^	9.2±9.7*^†^

Values shown are Mean ± SD. *P < 0.05 vs. SR values. **^†^** *P* < 0.05 vs. pacing at RA values. **^‡^** *P* < 0.05 vs. pacing at HB values. SR: sinus rhythm; RA: right atrium; RVA: right ventricular apex; RVOT: right ventricular outflow tract; HB: His bundle; LVA: left ventricular apex; LVS: left ventricular septum; RARV: right atrium and right ventricular apex; HR: heart rate; LVEF: left ventricular ejection fraction; LVEDV: left ventricular end diastolic volume; LVESV: left ventricular end systolic volume; MAPSE: mitral annular plane systolic excursion; TAPSE: tricuspid annular plane systolic excursion; LVESP: left ventricular end systolic pressure; LVEDP: left ventricular end diastolic pressure; TPR: time to peak rotation; TPT: time to peak twist; RSI: rotational synchronization index; aTPR: apical TPR; bTPR: basal TPR. R–R: R–R interval.

### LV Pressure and Function

Compared to pre-pacing and RA pacing, LVESP was significantly lower post ventricular pacing (all *P*<0.05). LVEDV, LVESV, LVEF, LVEDP, MAPSE and TAPSE were similar before and after pacing at various cardiac sites (Table 1).

### ECG

**Figure 2:**
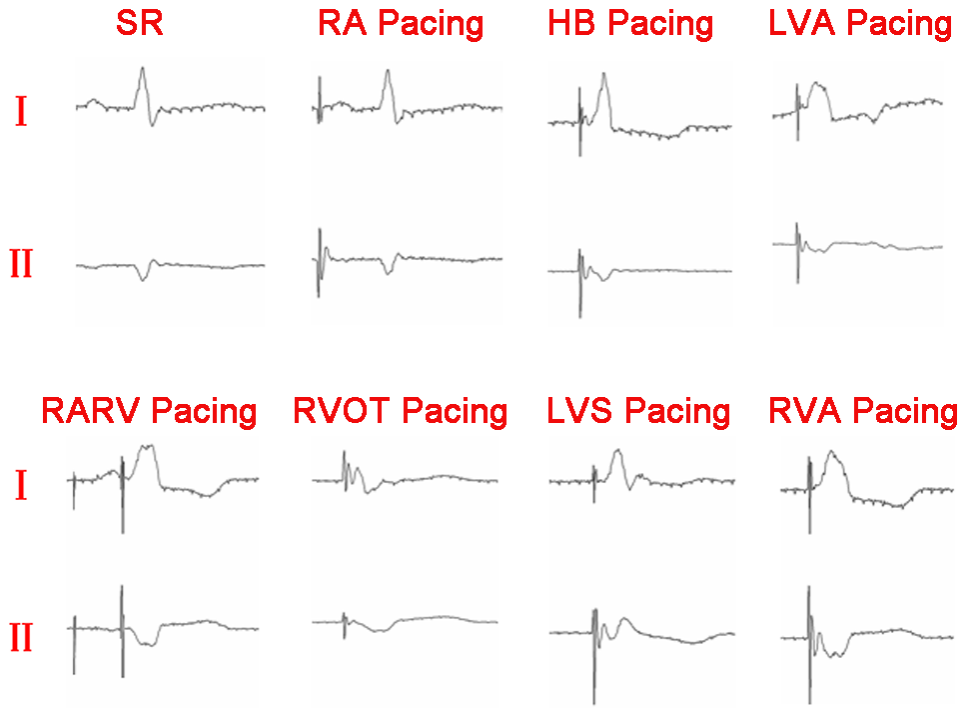
The QRS morphology of the same experimental dog at SR and different pacing side at I and II of ECG. As compared with HB and RA pacing, the QRS morphology was quite different at all involved ventricular pacing. SR: sinus rhythm; RA: right atrium; RVA: right ventricular apex; RVOT: right ventricular outflow tract; HB: His bundle; LVA: left ventricular apex; LVS: left ventricular septum; RARV: RA and RVA.

**Figure 3:**
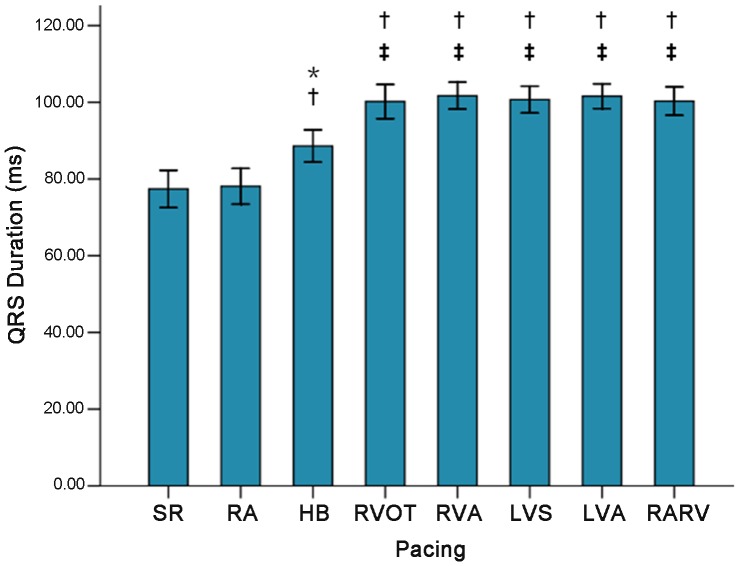
The QRS duration of SR at different pacing side. * *P<*0.05 vs. SR; ^†^ *P<*0.05 vs. pacing at RA; ^‡^ *P<*0.05 vs. pacing at HB. SR: sinus rhythm; RA: right atrium; RVA: right ventricular apex; RVOT: right ventricular outflow tract; HB: His bundle; LVA: left ventricular apex; LVS: left ventricular septum; RARV: RA and RVA.

As compared with pre-pacing and pacing at HB and RA, ventricular pacing resulted in significant changes on QRS morphology (Figure 2). The QRS duration was longer post HB pacing compared to RA pacing and pre-pacing, but shorter than post ventricular pacing (all *P*<0.05) (Figure 3).

### Rotation and Twist

**Figure 4:**
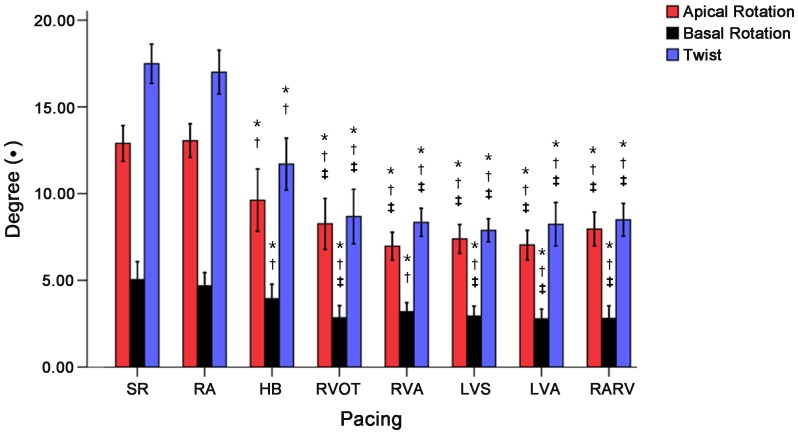
Changes in parameters of apical rotation, basal rotation and twist during different cardiac pacing and SR (Special Note: the basal dates are its absolute value). * *P<*0.05 vs. SR; ^†^ *P<*0.05 vs. pacing at RA; ^‡^ *P<*0.05 vs. pacing at HB. SR: sinus rhythm; RA: right atrium; RVA: right ventricular apex; RVOT: right ventricular outflow tract; HB: His bundle; LVA: left ventricular apex; LVS: left ventricular septum; RARV: RA and RVA.

The apical and basal rotation and twist values were significantly lower post HB pacing compared to RA pacing and pre-pacing, but these values were significantly larger post HB pacing than post left and right ventricular pacing (all *P*<0.05, expect basal rotation value post RVA pacing) (Figure 1, Figure 4).

### TPT, TPR and RSI

**Figure 5:**
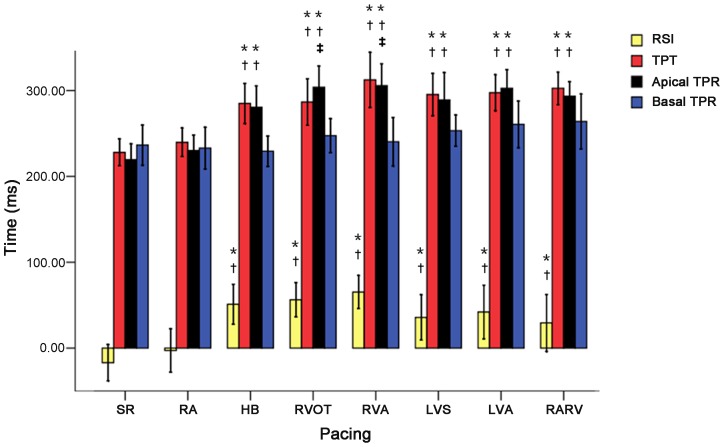
Changes in parameters of apical TPR, basal TPR, TPT, and RSI during different cardiac side pacing and SR. * *P<*0.05 vs. SR; ^†^ *P<*0.05 vs. pacing at RA; ^‡^ *P<*0.05 vs. pacing at HB. SR: sinus rhythm; RA: right atrium; RVA: right ventricular apex; RVOT: right ventricular outflow tract; HB: His bundle; LVA: left ventricular apex; LVS: left ventricular septum; RARV: RA and RVA; TPR: time to peak rotation; TPT: time to peak twist; RSI: rotational synchronization index.

TPT, aTPR and RSI were significantly longer post various ventricular pacing than those at pre-pacing and RA pacing (all *P<*0.05), and aTPR value was longer post RVOT and RVA pacing compared to post HB pacing (both *P*<0.05, Figure 1, Figure 5).

The above-mentioned statistical significance of TPT, aTPR, and RSI did not change after R–R interval adjustment. The pre-pacing bTPR value was smaller than that post pacing from all other cardiac sites (all *P<*0.05), and the bTPR post HB pacing was also smaller than that post RARV and LVA pacing (both *P*<0.05) (Table 1).

### Correlation Analysis

The apical rotation and twist were positively correlated with LVESP (*r* = 0.321 and 0.433, respectively, both *P*<0.01), and negatively correlated with QRS duration (*r* = –0.546 and –0.631, respectively, both *P*<0.001). There was no correlation among LVEDP, LEVSP, LVEDV, LVESV, LVEF, MAPSE and TAPSE with rotation or twist parameters. The twist was negatively correlated with TPT and RSI (*r* = –0.585 and –0.379, both *P*<0.001).

### Reproducibility Analysis

The calculated sample Pearson correlation coefficient was *r* = 0.836 at twist and 0.879 at TPT (both *P*<0.01) for results evaluated from two observers.

## Discussion

Present study provides some insight on the acute effects of pacing at different cardiac sites on LV twist and rotation. The major finding of this study is as follows: all ventricular pacing not only acutely reduced LV rotation and twist, but also increased TPR, TPT, and rotational dyssynchrony, while the negative impacts of RA pacing and HB pacing on LV twist and rotation are not that significant compared to ventricular pacing. Moreover, reduced rotation and twist post ventricular pacing is correlated with the reduced LVESP and prolonged QRS duration.

### Current Study on LV function post Cardic Pacing

Since conventional RVA pacing had significant deleterious effects on LV function, recent efforts are made on searching for alternative pacing site, such as RVOT, HB, LVS, RARV, and bi-ventricular pacing. Several studies already reported promising results on maintaining LV function by pacing from alternative cardiac sites [5], [9], [10], [17]. However, there were also controversial results on the advantage of those alternative pacing sites [18]–[20]. In addition, technical difficulties and concerns regarding lead positioning/stability and reliable long-term capture limit the clinical applicability of pacing from these specific cardiac sites [21]. Until now, there is still no consensus on the best and the most practical alternative pacing sites.

### Pacing Induced Change of Rotation and Twist

LV twist contributes significantly to LV function, myocardial shortening and thickening. During LV systolic phase, the base rotates in a clockwise direction, while the apex rotates in counterclockwise direction. LV twist is followed by rapid untwisting of the ventricle. The LV untwisting begins before aortic valve closure and before longitudinal and radial expansion (7,8). Because it is one of the earliest events leading to LV filling, untwisting may also be a critical determinant of early diastolic function [22]. Previous studies demonstrate that LV torsional mechanics, and LV twist/untwist are sensitive markers reflecting regional and global LV function changes [8], [23]. The peak of apical and basal rotation occurs almost synchronously in normal heart [8], [16]. However, in an abnormal condition, particularly due to altered LV electromechanical activation, the peaks of apical and basal rotation may be dyssynchronous [16], [24]. The dyssynchrony of apical and basal rotation could then reduce the LV twist, as shown in our results in that the twist was negatively correlated with RSI. Dyssynchrony of apical and basal rotation may also result in LV dysfunction [8], [24]. Because TPT and TPR are LV systolic parameters, the increase in TPT and TPR can correspondingly reduce the LV untwist and diastolic interval, which may subsequently induce LV dysfunction [25]–[28].

In the present study, we showed that pacing from ventricular sites could disturb the natural sequence of the LV electrical activity because of slowed myocardial activation, and thus result in reduced LV twist [29]. The negative impacts of RVA, RVOT, LVS, LVA and RARV pacing on QRS duration, QRS morphology and twist were significantly larger compared with those from RA pacing and HB pacing. Multiple underlying mechanisms might be responsible for the observed phenomenon. Theoretically, LV activation during HB pacing would be more similar to that during sinus rhythm, and HB pacing might thus lead to a relatively physiological electrical activation [30], [31]. However, HB pacing still resulted in unfavorable effects on LV twist in this study, although the negative impact on LV twist post HB pacing was somehow less than pacing from ventricular pacing. The reason may be that even the QRS morphology of HB pacing is quite similar with that during sinus rhythm at pre-pacing, the pacing site is just somehow close to the HB site but not exactly at the HB site. This might mean we might see effects of “Para-Hisian pacing” instead an exact “HB pacing”, so, HB pacing might still result in “pre-excitation” of a small region of inter ventricular septum and responsible for the observed negative impact on LV twist post HB pacing [32], [33].

Our results also demonstrated that RA pacing resulted in relatively less reduction on LV rotation and twist. LV rotation and twist values and LVESP were similar between post RA pacing andat the pre-pacing, which were much better than those post-pacing from ventricular sites. This might be explained by the fact that the RA pacing did not significantly affect electrical and mechanical activation of LV, as shown by a previous study [2]. It is to note that RA pacing is only recommended in sinus node disease and as a second choice after dual-chamber pacing (DDDR) with AV delay management based on the results of the DANPACE trial, since DANPACE trial results showed higher incidence of atrial fibrillation among patients with single-lead atrial pacing (AAIR) than in patients with DDDR [34]. Therefore, despite the mildest impact on acute LV mechanical and functional parameters post RA pacing, AAIR pacing mode has only very narrow niche in clinical practice. Similarly, despite the better LV mechanics post HB pacing than ventricular pacing, it is again difficult to get a stable position of the HB pacing lead clinically [21].

### Clinical Implication

LV twist/untwist, in addition to radial thickening and longitudinal shortening, are important elements responsible for effective LV pumping function. LV twist/untwist may be considered as a sensitive marker reflecting acute cardiac function changes in disease conditions. Thus, LV twist/untwist quantification might be helpful in monitoring early cardiac function changes or therapy effects post various therapy strategies (7,8). In our study, we showed that the time parameters of twist and rotation, such as TPT, TPR and RSI, were also changed as twist and rotation at different pacing. Therefore, results from the present study may indicate that measuring time parameters (TPT, TPR and RSI) might contribute to a better understanding of LV dyssynchrony induced by pacing at various cardiac sites,and to detect subclinical cardiac changes in some cases. Because it may be not easy to detect the acute effect of pacing on LV movement and function by using the conventional methods [35]–[37], twist and torsion as well its time parameter might be a good alternative for evaluating the acute impact of pacing on LV mechanics and function, which might be useful on finding and determining the best pacing sites, or on finding the suitable bivent pacing electrodes position and monitoring the therapeutic effects of cardiac resynchronization therapy [6].

### Study Limitations

Several limitations exist in our study. First, the pacing rates we used were relatively fast, like inthe previous studies [38]. In daily clinical practice, such high pacing rates are seldom used. However, higher pacing rate might mimic an exercise challenge. Second, we were surprised to find that the other data, such as LVEDP, LVESV, LVEF, MAPSE and TAPSE, remained unchanged acutely post pacing, while related changes were reported by other groups [29]. One of the reasons might be that longer time is needed to induce changes on the above-mentioned parameters [36], [39]. The dissatisfactory image quality of the apical 4 chamber views was also the reason that we did not analyze the LV strain. Third, these results were derived from an acute study protocol on dogs without structural heart disease, which may be different from the structural heart disease seen in clinical practice. Future studies are warranted to explore the impact of pacing on various cardiac sites on animal models with pre-existing structural cardiac diseases. Fourth, we did not compare the RVS pacing with SR because RVS is very close to HB or RVOT, and cannot readily be discriminated in dogs. Unfortunately, the LV pressure data were acquired by a fluid-filled catheter, and not by a high-fidelity conductance Millar® catheter. Therefore, dP/dt max and min, and indices of diastolic function like pressure half-time and the isovolumetric relaxation constant tau could not be determined. A correlation of these parameters with early diastolic de-rotation and de-twisting could increase the value of the manuscript, even in healthy animals.

## Conclusions

During cardiac pacing, a decrease in the apical and basal rotation and twist, a delay of apical TPR and TPT, and an increase in rotational dyssynchrony as well as decreased LVESP are observed with all ventricular pacing. HB and RA Pacing results in less negative effects on LV twist and rotation compared to other ventricular pacing in healthy dogs.
